# Credit to the Fruit Fly: How the Tiny Insect Lights Up Our Understanding of Human Disease

**DOI:** 10.3390/insects17070681

**Published:** 2026-06-30

**Authors:** Yansong Zhang, Yao Wang, Yizhi Li, Alan Jian Zhu, Min Liu

**Affiliations:** 1 State Key Laboratory of Membrane Biology, Beijing Advanced Center of RNA Biology, School of Life Sciences, Peking-Tsinghua Joint Center for Life Sciences, Peking University, Beijing 100871, China; 2 Peking University Chengdu Academy for Advanced Interdisciplinary Biotechnologies, Chengdu 610213, China

**Keywords:** *Drosophila melanogaster*, insect biology, disease model, molecular mechanism, translational research

## Abstract

*Drosophila melanogaster*, widely known as the fruit fly, has emerged as a pivotal model organism for studying development and signaling transduction. Its fully sequenced genome, short generation time, and powerful genetic toolkit—including the Gal4/UAS system, RNA interference, and CRISPR-Cas9—enable precise, tissue-specific manipulation and high-throughput functional analyses. Despite differences in anatomy, the internal organ systems of *Drosophila melanogaster*, including the nervous system, heart, fat body, oenocytes, and nephrocytes, exhibit conserved molecular pathways and physiological functions comparable to those of humans. The morphological differences between invertebrates and vertebrates have long led researchers to undervalue the studies of insects in underlying the pathogenesis of human diseases. Over the past decades, the fruit fly has been widely validated for modeling the pathogenesis of neurodegenerative, cardiovascular, metabolic, renal, and muscular disorders. In this review, we systematically summarize the conserved molecular pathways and organ functions between the fruit fly and human, and provide examples of recent studies that use the fruit fly as a model system to answer questions associated with human diseases. We also discuss how *Drosophila* help researchers to fulfill the gap from mechanistic study toward translational research, and provide methodological considerations regarding the utility of *Drosophila* models in drug screening.

## Simple Summary

*Drosophila melanogaster*, commonly known as the fruit fly, is a flagship model organism for studying the molecular mechanisms underlying development and many physiological processes. The internal organ systems of *Drosophila melanogaster*, including the nervous system, heart, fat body, oenocytes, and nephrocytes, share conserved molecular pathways and physiological functions with humans. The genetic tractability, short generation time, powerful toolkit, in combination with advanced approaches such as single-cell, spatial multi-omics and optogenetic systems, solidifies *Drosophila* as an indispensable model in biomedical research. For a long time, the marked morphological differences between invertebrates and vertebrates have led researchers to underestimate the value of insects in elucidating the pathogenesis of human diseases. In the past decades, the fruit fly has been widely validated as a powerful model for investigating the pathogenesis of neurodegenerative, cardiovascular, metabolic, renal, and muscular disorders. This review systematically summarizes the conserved molecular pathways and analogous organ functions shared between insects and humans, and highlights recent studies that have used the fruit fly as a model system to address questions related to human disease. We further discuss how *Drosophila* helps bridge the gap between mechanistic studies and translational research, with particular attention to methodological considerations for its application in drug screening.

## 1. Introduction

**Figure 1:**
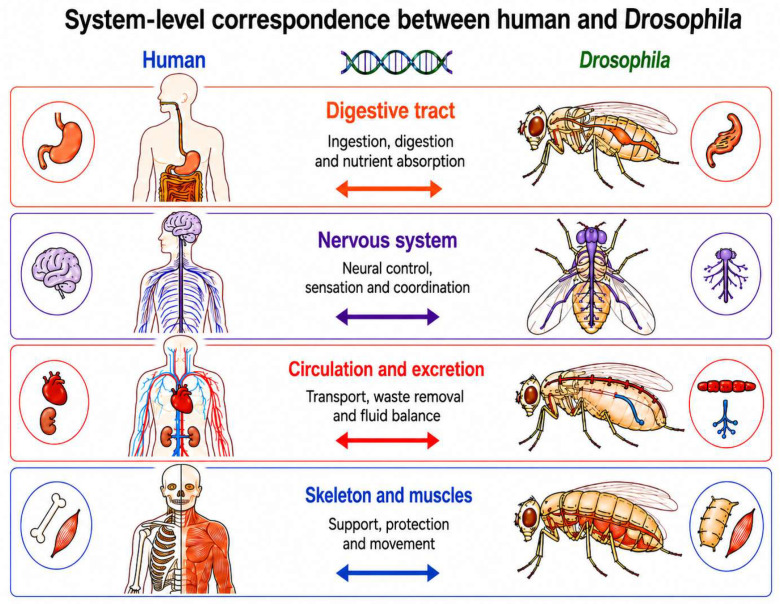
System-level organ correspondence between human and *Drosophila*. Schematic comparison highlighting conserved organ systems and their primary functions. The human and *Drosophila* organs are grouped by system, including digestive tract, nervous system, circulation and excretion, as well as skeleton and muscles. Despite anatomical differences, key physiological roles are evolutionarily conserved between human and *Drosophila*.

*Drosophila melanogaster*, commonly known as the fruit fly, has long served as a cornerstone of modern genetics, highlighting the power of insect models in uncovering fundamental biological principles (Figure 1). Its exceptional genetic tractability, combined with a fully sequenced genome encompassing over 13,900 protein-coding genes, provides a versatile platform for molecular, developmental, and physiological studies. Research in this model organism has revealed and characterized numerous evolutionarily conserved signaling pathways, including Hedgehog, Wingless/Wnt, Notch, Hippo, JAK-STAT, and insulin/TOR. These conserved molecular mechanisms, together with a short life cycle and high fecundity, allow sophisticated in vivo genetic screens and functional analyses that are difficult to achieve in larger organisms. Leveraging these advantages, *Drosophila* has become an indispensable model for dissecting conserved pathways underlying both insect physiology and human disease. Studies in this organism have provided mechanistic insights into inter-organ communication and systemic metabolic regulation, establishing a predictive framework for modeling the pathogenesis of neurodegenerative, cardiovascular, metabolic, renal, and muscular disorders. The combination of evolutionary conservation, experimental tractability, and compatibility with cutting-edge technologies positions *Drosophila* as a versatile platform bridging fundamental discovery and translational medicine. These attributes underscore its enduring relevance in contemporary biomedical research.

## 2. Genetic Tractability and Physiological Conservation: The Foundation of *Drosophila* as a Disease Model

*Drosophila melanogaster* has served as an important model organism in biomedical research for over a century [1,2,3]. Its enduring value stems from the remarkable evolutionary conservation shared with humans at the molecular, cellular, and physiological levels. Comparative genomic analyses indicate that approximately 75% of human genes associated with disease have clear homologs in *Drosophila* [4,5]. This conservation encompasses not only individual gene sequences but also signaling networks, organelle functions, and coordinated inter-organ regulation. Core pathways controlling cell growth and proliferation—such as Hippo, JAK-STAT, Wnt/β-catenin, and insulin/TOR—retain nearly identical components and functional logic between flies and mammals [6,7,8,9,10].

Although the *Drosophila* nervous system is structurally simpler than that of vertebrates, its neurons exhibit conserved electrophysiological properties, synaptic transmission mechanisms, and blood–brain barrier functions [11,12,13,14]. Similarly, the fat body performs roles analogous to the human liver and white adipose tissue, managing nutrient storage, metabolic regulation, and hormone secretion [15,16,17]. Malpighian tubules and nephrocytes mirror the functions of mammalian renal tubules and podocytes, executing osmoregulation and waste filtration [18]. These conserved organ-level functions enable *Drosophila* to model systemic metabolic disturbances and inter-organ communication relevant to human disease [19].

Beyond evolutionary conservation, the exceptional experimental tractability of *Drosophila* is a key factor underpinning its utility as a disease model. The Gal4/UAS system allows precise, cell-type-specific manipulation of gene expression, enabling targeted overexpression or knockdown in specific tissues [20,21,22]. CRISPR-Cas9 technology further facilitates the routine introduction of human disease-associated point mutations or gene knockouts [23]. A broad array of transgenic reporters supports monitoring of cellular processes, including apoptosis, autophagy, and mitochondrial dynamics, while live imaging and behavioral assays provide powerful phenotypic readouts [23,24]. Combined with a short life cycle and high reproductive capacity, these features permit rapid, large-scale genetic screens that are difficult to achieve in mammalian models [25]. Practically, *Drosophila* genetic screens usually start from a robust disease-relevant phenotype that can be rapidly and reproducibly scored, including viability, morphology, locomotor activity, lifespan, tissue degeneration, reporter activity, or tumor growth [26,27]. The disease model is then crossed to genome-wide or candidate RNAi, CRISPR, deficiency, mutant, or overexpression libraries to identify genetic perturbations that suppress or enhance the phenotype [28,29]. Subsequent hit prioritization should consider phenotypic strength, reproducibility, human orthology, and pathway relevance, followed by secondary validation using independent RNAi or sgRNA lines, appropriate genetic controls, rescue experiments, and orthogonal assays to minimize off-target, developmental, or background effects [30,31]. Collectively, these advantages have transformed *Drosophila* from a classical developmental biology organism into a versatile platform for studying adult-onset and complex human diseases.

## 3. Neural Circuitry and Proteostasis: Decoding the Pathogenesis of Neurodegeneration

Neurodegenerative diseases, including Alzheimer’s disease, Parkinson’s disease, Huntington’s disease, and amyotrophic lateral sclerosis, are characterized by the progressive loss of specific neuronal populations [32,33]. *Drosophila* models have been instrumental in recapitulating key cellular and molecular features of these disorders, providing mechanistic insights into their complex pathogenesis [34,35]. Because these disorders are typically age-dependent, most *Drosophila* models of neurodegenerative diseases are established and phenotypically assessed in adult flies, using readouts such as progressive locomotor decline, shortened lifespan, neuronal loss, and disease protein aggregation; larval models are used more selectively for neurodevelopmental phenotypes, neuromuscular junction defects, or rapid behavioral screening [36,37].

### 3.1. Alzheimer’s Disease

In Alzheimer’s disease research, *Drosophila* models are widely employed to study the toxicity of β-amyloid (Aβ) and hyperphosphorylated Tau [38,39,40]. Expression of human Aβ42 in flies leads to age-dependent learning and memory deficits, neuronal loss, and extracellular aggregates resembling senile plaques [41,42]. Notably, oligomeric Aβ intermediates, rather than mature plaques, primarily disrupt synaptic function by activating stress signaling pathways such as c-Jun N-terminal kinase (JNK), ultimately inducing apoptosis [43,44,45]. Using the visual and olfactory circuits as quantitative readouts, studies have demonstrated that Aβ selectively impairs cholinergic neurons and the mushroom body—the fly’s learning and memory center—offering mechanistic insight into early cognitive decline [46].

Tauopathies are modeled by expressing human mutant Tau in *Drosophila*, producing retinal degeneration, motor deficits, and shortened lifespan [38,47,48]. Hyperphosphorylated Tau loses its microtubule-stabilizing function, leading to axonal transport defects, while simultaneously acquiring a toxic gain-of-function, forming neurofibrillary tangle-like aggregates [49,50,51]. Genetic screens have revealed that reducing glycogen synthase kinase-3β (GSK-3β) activity or enhancing protein phosphatase 2A (PP2A) activity mitigates Tau-induced neurodegeneration and behavioral deficits [34,35]. Small-molecule screens in these models, such as the kinase inhibitor Ro 31-8220, have successfully reduced Tau phosphorylation, improving memory and motor performance in flies, with translational validation in human neuroblastoma cells [52].

### 3.2. Parkinson’s Disease and Mitochondrial Quality Control

*Drosophila* models of Parkinson’s disease recapitulate familial disease caused by mutations in PINK1 and Parkin, displaying progressive motor impairment, selective dopaminergic neuron loss, and abnormal mitochondrial morphology [53,54,55]. The PINK1/Parkin pathway is central to mitochondrial quality control: upon mitochondrial damage, PINK1 stabilizes on the outer membrane and recruits Parkin, which ubiquitinates outer membrane proteins to initiate mitophagy via autophagy receptors [56,57,58]. Additionally, basal mitophagy occurs continuously in most tissues independent of PINK1/Parkin, suggesting compensatory mechanisms [59,60]. Recent behavioral screening in *Drosophila* identifies molecular Parkinson’s disease subgroups, revealing distinct pathways and potential therapeutic targets across genetic backgrounds [3]. Additionally, environmental toxin-induced models, using paraquat or rotenone, replicate sporadic Parkinson’s disease features and allow the study of gene-environment interactions [61,62]. For instance, glial-specific knockdown of the RNA-binding protein Spen increases sensitivity to paraquat and disrupts lipid droplet metabolism, highlighting glial contributions to neuroprotection [63,64].

### 3.3. Polyglutamine Diseases and Other Neurological Disorders

For polyglutamine (polyQ) expansion diseases, including Huntington’s disease and spinocerebellar ataxias, *Drosophila* expressing expanded polyQ protein fragments recapitulates nuclear inclusions, progressive neuronal degeneration, and motor deficits [65,66]. Aggregated polyQ proteins sequester transcription factors like CREB-binding protein (CBP), inducing global transcriptional dysregulation and interfering with the ubiquitin-proteasome and autophagy-lysosome pathways [66,67,68,69,70]. Upregulation of molecular chaperones (e.g., Hsp70) or pharmacological activation of autophagy (e.g., rapamycin) reduces aggregation and toxicity, identifying potential therapeutic targets [71,72]. Models expressing mutant ataxin-1, -3, and -7 have clarified interactions among protein aggregation, impaired degradation, altered gene expression, and mitochondrial dysfunction in disease pathogenesis [34,73].

Beyond polyQ diseases, *Drosophila* has advanced research on neurodevelopmental disorders (e.g., Fragile X and Rett syndromes) and motor neuron diseases such as amyotrophic lateral sclerosis. Expression of mutant human SOD1 or TDP-43 leads to motor neuron degeneration, muscle denervation, and impaired locomotion, providing platforms for genetic modifier screens and therapeutic testing [74,75,76,77,78,79].

## 4. Metabolic Physiology and Nutrient Sensing: Insights into Inter-Organ Communication and Metabolic Disorders

**Figure 2:**
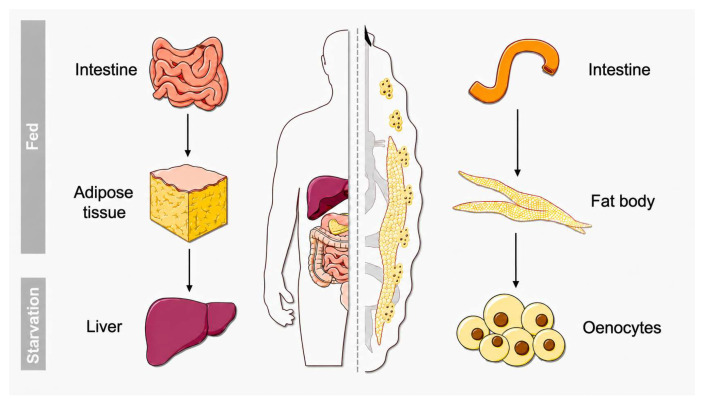
Comparative lipid flow between human and *Drosophila* under fed and starvation conditions. Illustration of energy storage and mobilization in human (left) and *Drosophila* (right). In the fed state, lipids are absorbed from the intestine and stored in adipose tissue (human) and fat body (*Drosophila*). Under starvation, lipid is mobilized from adipose tissue and fat body toward liver (human) and oenocytes (*Drosophila*). This model emphasizes the functional analogy of inter-organ metabolic regulation between these two species.

Metabolic diseases, including obesity, diabetes, and metabolic dysfunction-associated steatotic liver disease (MASLD), represent major global health challenges characterized by systemic disruptions in energy homeostasis. *Drosophila* possesses a metabolic regulatory system highly conserved with mammals (Figure 2), making it an ideal model to dissect the complex mechanisms underlying these disorders [15]. In this section, we focus primarily on larval-stage metabolic models, because larvae undergo rapid growth and display highly dynamic changes in insulin/TOR signaling, fat-body expansion, and lipid accumulation. These life-stage-specific features are experimentally advantageous: juvenile larvae can show pronounced fat accumulation in the fat body, which can be readily quantified by buoyancy-based assays, whereas adult lipid storage is more tightly controlled and depends more heavily on circulating triglycerides and adult fat-body homeostasis [80,81].

### 4.1. Energy Metabolism Regulation and Obesity/Diabetes Models

In the fruit fly, energy storage primarily occurs in the fat body, which integrates functions of the liver and adipose tissue [15]. Insulin-like peptides, secreted by insulin-producing cells in the brain, function analogously to mammalian insulin, while the cytokine Unpaired 2 (Upd2) from the fat body acts as a functional leptin homolog [82,83,84]. Together, these signals form a conserved feedback loop regulating systemic energy status [85]. Exposure to high-sugar or high-fat diets induces phenotypes resembling human metabolic syndrome, including triglyceride accumulation, hyperglycemia, and insulin resistance [86]. High-fat diets disrupt conserved insulin/TOR signaling in the myocardium, resulting in cardiac lipid deposition and impaired contractility, mirroring diabetic cardiomyopathy [87,88]. Interventions targeting TOR signaling or enhancing myocardial lipolysis can reverse these cardiotoxic effects [88,89]. Research on the fat–brain axis has further revealed intricate regulatory mechanisms. Upd2 signals to energy-sensing neurons, modulating inhibitory synapse number to establish a permissive neural tone for insulin release. Conversely, insulin signaling feedback adjusts synapse density to maintain homeostasis. This bidirectional regulation constitutes a fine-tuned loop essential for maintaining fat storage equilibrium [11,90].

### 4.2. Mechanisms and Therapeutic Targets of MASLD

*Drosophila* has contributed significantly to MASLD research. Dietary essential amino acids, particularly leucine and isoleucine, activate the E3 ubiquitin ligase Ubr1, promoting K48-linked polyubiquitination and proteasomal degradation of Plin2, a lipid droplet surface protein, thereby reducing lipid accumulation. This mechanism is conserved in flies, mice, and human hepatocytes, suggesting therapeutic potential via Ubr1 activation [91,92]. Oxidative stress also impacts lipid droplet metabolism. The lipid peroxidation product 4-hydroxynonenal (4-HNE) modifies Hsc70-4, inhibiting Ubr1 activation and stabilizing Plin2, leading to protective lipid droplet accumulation. However, in glioma, elevated 4-HNE modification of HSPA8, the human Hsc70-4 homolog, correlates with tumor malignancy, suggesting pathological hijacking of this pathway [93]. Targeting *Plin2* via siRNA in flies and mice alleviates steatosis, and a chemically modified GalNAc-si*Plin2* has shown safety and efficacy in mice and entered clinical evaluation [94]. These studies exemplify a complete translational pipeline from *Drosophila* mechanistic discovery to mammalian validation and clinical application.

### 4.3. Gut–Brain Axis and Feeding Behavior Regulation

*Drosophila* also serves as a powerful model to study the gut–brain axis and feeding behavior [95,96]. Enterocytes in the R2 intestinal region sense dietary essential amino acid (EAA) deficiency and induce the neuropeptide CNMa, which signals the brain via its receptor to promote selective appetite for EAA-rich foods. Symbiotic microbes can supply some EAAs, buffering this signal, and alterations in microbial composition directly influence host nutrient preference [97]. A high-protein diet triggers gut-derived CCHamide1 secretion, which activates sNPF neurons, coordinating protein-specific satiety, feeding behavior, and systemic metabolic homeostasis, highlighting a gut–brain axis regulatory mechanism [98]. Additionally, high-sugar diets disrupt microbiota homeostasis, such as reducing *Acetobacter*, triggering intestinal inflammation that alters central neurotransmitter balance and results in neurobehavioral deficits, including reduced sleep. Genetic interventions targeting inflammatory factors or probiotic supplementation can restore normal physiology, highlighting the therapeutic potential of modulating the gut-inflammation-brain axis [99,100,101]. These findings demonstrate how the intestine integrates nutritional signals and engages in bidirectional communication with the brain to regulate systemic physiology and feeding behavior at molecular and cellular levels.

### 4.4. Comparative Insect Models for Metabolic Endocrinology

Although *Drosophila melanogaster* remains the most genetically tractable insect model for metabolic disease research, recent work has increasingly emphasized the value of non-model insects for addressing questions that are difficult to capture in flies alone. Insect endocrinology has a long history extending beyond *Drosophila*: many neuropeptides and peptide hormones were originally discovered or functionally characterized in diverse insect species, and comparative studies have revealed both conserved and lineage-specific strategies for coordinating growth, feeding, reproduction, and energy storage [102]. Thus, *Drosophila* should be viewed as a central genetic platform within a broader comparative framework rather than as a complete representation of insect metabolic diversity.

Mosquitoes provide a particularly informative system for studying the coupling between nutrition, insulin-like peptide (ILP) signaling, and reproduction. Female mosquitoes shift from carbohydrate feeding to a protein-rich blood meal, which activates insulin/TOR signaling, blood digestion, vitellogenesis, and egg maturation [103,104]. In *Aedes aegypti*, multiple ILPs and ovary ecdysteroidogenic hormone are released or regulated during the gonadotropic cycle and differentially stimulate nutrient storage, insulin-pathway activation, and egg formation [105]. This blood-meal-dependent reproductive switch provides an experimentally powerful model for studying how nutrient identity and endocrine signaling coordinate metabolism with reproductive investment.

Coleopteran beetles and other insects further expand the comparative value of insect metabolism models because several species possess multiple insulin receptors, whereas *Drosophila* has only one canonical insulin receptor. In the red flour beetle *Tribolium castaneum*, two insulin receptor genes have functionally diverged in development and reproduction, offering opportunities to examine receptor specificity, tissue expression, and age-dependent endocrine regulation in a manner that may more closely resemble the receptor diversification seen in mammals [106]. More broadly, phylogenetic analyses indicate that insect insulin receptor multiplicity and decoy receptor evolution are widespread and functionally significant [107]. These non-*Drosophila* systems therefore complement fly genetics by broadening the evolutionary and physiological context for studying insulin signaling, nutrient sensing, and metabolic disease mechanisms.

## 5. Reproductive System: Metabolic Integration, Sex Differences, and Disease Relevance

The reproductive system of *Drosophila melanogaster* provides a genetically tractable model for studying how stem-cell niches, systemic metabolism, sex-specific signaling, and aging regulate reproductive capacity [108,109,110]. Here, we primarily discuss adult gonads, as spermatogenesis, oogenesis, reproductive output, stem-cell niche maintenance, and gonadal aging are usually analyzed in adult testes and ovaries, whereas embryonic or larval gonads are mainly used for early gonad development and sex-determination studies [109]. Although flies cannot fully recapitulate the mammalian hypothalamic–pituitary–gonadal axis, their gonads reveal conserved principles relevant to infertility and reproductive decline. In the testis, hub-derived Unpaired/JAK/STAT signaling maintains germline and cyst stem cells, while adhesion and soma–germline communication support spermatogenesis [111,112,113]. In the ovary, cap-cell-derived BMP/Dpp signaling maintains female germline stem cells (GSCs), whereas follicle cells regulate oocyte polarity and egg chamber development [114,115].

Reproduction in *Drosophila melanogaster* is tightly coupled to systemic metabolic state because gametogenesis, vitellogenesis, mating, and egg production are energetically demanding [116,117]. In females, insulin/IGF-like peptide (ILP) signaling promotes vitellogenesis and yolk uptake, whereas dietary restriction or impaired insulin signaling arrests oogenesis before vitellogenic stages [118,119]. Recent studies further indicate that female reproductive output is coordinated by communication among the fat body, germline, follicle cells, and neuroendocrine circuits [120,121,122]. Integrated stress response signaling in fat tissue acts as a nutrient-sensitive metabolic sensor regulating yolk lipoprotein production, oocyte maturation, and ovulation [117,123].

### 5.1. Metabolic Integration and Sex-Specific Regulation of Reproduction

Within the ovary, metabolic control operates through mitochondrial, small-RNA, and lipid pathways. Nutrient-dependent regulation of the stable intron sisR-1 modulates mitochondrial quality control and mitophagy during oogenesis, thereby affecting oocyte quality after fasting [124]. piRNAs and the PIWI protein Aubergine promote glycolytic reprogramming in female germline stem cells, extending the role of small-RNA pathways beyond transposon repression [125]. Acetyl-CoA carboxylase maintains energetic balance during oogenesis by coordinating fatty-acid metabolism, TOR signaling, intracellular trafficking, fusome organization, and oocyte fate [116]. Follicle cells provide another regulatory layer, as JAK/STAT-dependent contact between posterior follicle cells and the oocyte defines the oskar mRNA anchoring region and influences embryonic germline development [126].

Male reproduction is also metabolically regulated. In the testis, EGFR-stimulated autophagy supports cyst stem cell maintenance and lipid homeostasis, whereas mitochondrial fission limits ROS-dependent aberrant EGFR activation and premature germline differentiation [127,128]. Sex-specific regulation through tra, dsx, fru, and Sxl further shapes reproductive and metabolic physiology, and cellular sex can act throughout the organism to generate tissue-intrinsic sexual dimorphism [129].

### 5.2. Relevance to Human Reproductive and Sex-Biased Diseases

The *Drosophila* reproductive system provides mechanistic insight into infertility, reproductive aging, metabolic reproductive dysfunction, gonadal pathologies, and sex-biased disease susceptibility [130,131]. In males, the testis niche models how defective somatic support, impaired adhesion, disrupted soma–germline communication, mitochondrial dysfunction, oxidative stress, and epigenetic instability compromise spermatogenesis [130,132,133]. Loss of SOCS36E enhances JAK/STAT and MAPK signaling in cyst stem cells, increases βPS-integrin-mediated adhesion to the hub, and allows cyst stem cells to outcompete germline stem cells, leading to germline loss [134,135]. Polycomb group genes such as Psc and Su(z)2 maintain somatic cyst cell identity; their disruption induces somatic cyst cell tumors that non-autonomously displace germline cells [136].

The ovary is particularly useful for studying female infertility and oocyte-quality decline. Oocyte maturation depends on insulin signaling, lipid balance, mitochondrial quality control, translational regulation, and follicle-cell support [119,137,138]. Findings that stable introns modulate mitophagy and oocyte quality, piRNA pathways regulate germline stem-cell glycolysis, and acetyl-CoA carboxylase coordinates metabolic and structural programs during oogenesis provide entry points into mechanisms relevant to human oocyte competence [125]. Although flies cannot fully model mammalian endocrine syndromes such as polycystic ovary syndrome, they can dissect conserved metabolic components of reproductive dysfunction, including insulin resistance-related defects, lipid imbalance, mitochondrial stress, and impaired germline–somatic communication [117,121,139,140].

Fly models also clarify reproductive aging as a tissue-level process. Reproductive diapause preserves female germline stem cells, whereas ovarian niche aging involves transcriptional and alternative-splicing changes in support cells [141,142]. In males, aging reduces hub-cell DE-cadherin and Unpaired expression, correlating with germline stem-cell loss [143]. Together, these studies show how sex, metabolism, stem-cell niches, and reproductive state interact to shape disease susceptibility [129].

## 6. Oncogenic Signaling and Tumor-Host Interactions: From Conserved Mechanisms to Cancer Cachexia

Although *Drosophila* does not spontaneously develop solid tumors identical to those in humans, its sophisticated genetic tools allow precise activation of oncogenes (e.g., *ras*, *src*) or inactivation of tumor suppressor genes (e.g., *scribble*, *lgl*, *dlg*) in specific tissues, generating tumors with invasive and metastatic features [5,144,145,146,147,148]. These models provide unique opportunities to study tumorigenesis, progression, and tumor-host interactions in vivo. Most classical epithelial tumor models, including Ras(V12) combined with loss of *scribble*, *lgl*, or *dlg*, are generated in larval imaginal discs, where rapid tissue growth facilitates analysis of tumor overgrowth, invasion, systemic wasting, and tumor–host interactions; in contrast, adult flies are more commonly used for intestinal stem-cell-driven tumors, age-associated epithelial dysplasia, and long-term effects of tumor burden [149].

### 6.1. Mechanisms of Tumorigenesis and Progression

*Drosophila* research has significantly advanced our understanding of the Hippo signaling pathway, a critical tumor suppressor network. Loss of core Hippo components (e.g., *Hpo*, *Wts*, *Sav*, *Mats*) induces tissue overgrowth [150,151], while aberrant activation of its downstream effector Yorkie (Yki, homolog of YAP/TAZ) promotes cell proliferation and survival autonomously and triggers paracrine signals, including JAK-STAT activation via secreted cytokines, driving tumor-like growth [152,153,154]. For instance, Yki activation in intestinal stem cells induces excessive proliferation and upregulation of the insulin/IGF antagonist ImpL2, establishing a direct link between local tumor growth and systemic metabolic alterations [155,156,157].

Tumor heterogeneity and intercellular cooperation have also been elucidated using *Drosophila* models. In larval eye imaginal discs, tumor cells with Ras^V12^ activation and polarity gene loss secrete Pvf1 to recruit hemocytes. The hemocytes, in turn, release Spätzle, activating Toll receptors on tumor cells and promoting malignant transformation through the JNK-Hippo cascade in cooperation with Ras signaling [158,159]. Moreover, adipocyte lipid metabolism remotely modulates tumor growth by promoting Wnt5 secretion via Nplp2-associated lipoproteins, demonstrating how systemic metabolic states influence tumor progression at a distance [160]. Collectively, these studies underscore how tumor subpopulations communicate with their microenvironment and systemic metabolic cues to coordinate paracrine and endocrine signals, revealing cooperative dynamics that drive tumor progression.

### 6.2. Cancer Cachexia and Systemic Host Response

Cancer cachexia, a fatal systemic wasting syndrome, is difficult to study directly in patients. *Drosophila* tumor models have been instrumental in revealing its mechanisms. Malignant tumors secrete ImpL2, a homolog of insulin-like growth factor binding protein, which enters the circulation, inhibits host insulin-like peptides, and induces wasting of distal fat body, muscle, and gonads, accompanied by hyperglycemia-like metabolic abnormalities [155,161,162]. Knockdown of ImpL2 in tumors alleviates these systemic effects, identifying it as a key mediator of cachexia [161]. Additional factors, such as the pro-inflammatory cytokine Upd3 (IL-6 homolog) and TNF-α homolog Eiger, contribute to cachexia, highlighting the molecular heterogeneity of tumor-driven systemic wasting [163,164]. These models provide an invaluable platform for dissecting tumor-induced host reprogramming and identifying potential therapeutic targets.

### 6.3. Personalized Drug Screening Platform

*Drosophila* has also been leveraged as a platform for personalized medicine. In a pioneering study, a patient with metastatic colorectal cancer harboring a KRAS mutation underwent whole-genome tumor profiling, identifying nine key driver genes. Researchers constructed a personalized *Drosophila* tumor model incorporating these alterations for high-throughput drug screening. In practical terms, constructing a *Drosophila* “avatar” model generally involves prioritizing patient-specific driver alterations from tumor genomic profiling, mapping them to conserved fly orthologs or pathways, and modeling oncogene activation or tumor suppressor loss using UAS-driven transgenes, RNAi, CRISPR-based disruption, or available mutant alleles [165]. These alterations are then combined into a single genotype and expressed in a disease-relevant tissue, such as the adult hindgut for colorectal cancer, through tissue-specific and temporally controlled Gal4/Gal80 systems [166,167,168]. After confirming a robust phenotype, such as tumor overgrowth, epithelial disorganization, dissemination, lethality, or reporter activation, candidate drugs are screened as single agents or combinations, with hits prioritized according to phenotypic rescue, toxicity, reproducibility, mechanistic plausibility, and clinical actionability [169]. The combination of the MEK inhibitor trametinib and the bisphosphonate zoledronic acid significantly inhibited tumor growth in the fly model. Translating this regimen to the patient resulted in clinical remission of both target and non-target lesions, with sustained efficacy over months [5,166,170]. This work highlights the potential of *Drosophila* “avatar” models for rapid, patient-specific therapeutic screening, offering new avenues for precision oncology.

## 7. Muscle Development and Degeneration: Elucidating Mechanisms of Muscular Disorders

*Drosophila* models have proven invaluable for dissecting the complex pathological processes underlying muscle diseases, providing a clear mechanistic framework from cell-autonomous degeneration to systemic wasting. These studies have advanced understanding of primary muscle disorders, such as Duchenne muscular dystrophy (DMD), and offered new perspectives on secondary wasting syndromes, including cancer cachexia. Larval models are particularly useful for studying body-wall muscle development, neuromuscular-junction defects, and tumor-induced cachectic wasting, whereas adult flies are more suitable for modeling progressive muscle degeneration, flight or climbing impairment, and age-dependent muscle functional decline [171,172].

### 7.1. Primary Muscle Degeneration: Conserved Molecular Mechanisms

*Drosophila* faithfully models human muscular dystrophies. A seminal finding was the central role of the Dystroglycan–Dystrophin (Dg-Dys) complex in muscle maintenance. Loss of Dg or Dys function in flies leads to progressive, age-dependent muscle degeneration, motor impairment, and vacuolization, closely recapitulating the core human disease phenotype [173]. Beyond phenotypic mimicry, mechanistic studies revealed that the Dg-Dys complex is essential for proper neuromuscular junction function, with defects causing abnormal acetylcholine signaling and synaptic dysfunction [174,175]. Importantly, genetic interactions between Dg and the Insulin Receptor (InR) pathway link structural integrity to metabolic regulation, suggesting that metabolic dysregulation contributes to muscular dystrophy pathogenesis. Collectively, these findings establish *Drosophila* as a robust platform for investigating cell-autonomous mechanisms of muscle degeneration.

### 7.2. Secondary Systemic Wasting: Tumor-Host Interactions

*Drosophila* models have uniquely advanced understanding of cancer cachexia. Tumors induced in specific tissues, such as the eye imaginal disc or intestine, trigger severe wasting of distal tissues—including fat, muscle, and gonads—mirroring human cachexia. The model’s genetic tractability allows rapid identification of tumor-secreted factors mediating systemic wasting [157,161,176]. Different tumor genotypes secrete distinct mediators: some tumors release the insulin-like growth factor binding protein homolog ImpL2, antagonizing systemic insulin/IGF signaling, which enhances catabolism (e.g., autophagy) and inhibits anabolism [155,161,177,178]. Other tumors secrete matrix metalloproteinase Mmp1, promoting muscle atrophy via extracellular matrix disruption, acting in parallel with ImpL2 [179]. These studies confirm that insulin signaling resistance is a conserved core mechanism of cachexia and reveal additional pathogenic pathways, such as microenvironment-mediated tissue remodeling. *Drosophila* thus provides an efficient screening platform to identify conserved mediators of muscle wasting.

### 7.3. Translational Implications: From Mechanism to Therapy

*Drosophila* muscle models offer direct translational insights. In primary muscular dystrophy, they facilitate screening of modifier genes or therapeutic compounds, including those targeting pathways like sphingosine-1-phosphate (S1P) that may confer protection across multiple muscle-wasting conditions [180,181]. In cachexia, fly studies reinforce the notion that “cachexia is not a single disease,” emphasizing the need to dissect tumor-specific secreted factors and downstream pathways [155,161,182]. These mechanistic insights provide a foundation for developing personalized anti-cachexia therapies targeting specific tumor types or molecular pathways.

## 8. Renal Physiology in Insects: Modeling Human Kidney Structure, Function, and Disease

The *Drosophila* excretory system comprises Malpighian tubules, which are functionally analogous to mammalian renal tubules, the hindgut, and nephrocytes, which share characteristics of both podocytes and proximal tubule cells. Numerous human kidney disease-associated genes, including those implicated in nephrolithiasis, renal tubular acidosis, and nephrotic syndrome, have conserved homologs in *Drosophila* [183,184]. In the renal disease models discussed here, adult flies are most commonly used for functional analyses of Malpighian tubule physiology, nephrolithiasis, tumor-induced renal dysfunction, and nephrocyte filtration, whereas embryonic or larval stages are mainly used to study excretory organ development and early morphogenesis [185].

These models allow convenient in vivo investigation of gene functions in ion homeostasis, metabolic waste filtration, and reabsorption [183,186]. For instance, manipulating *Drosophila* homologs has enabled the development of models for calcium oxalate nephrolithiasis and polycystic kidney disease, permitting quantitative observation of stone formation and disease progression. Recent studies further demonstrate that tumor-induced secretion of the antidiuretic peptide isoform F of the ion transport peptide (ITP_F_) impairs renal-like Malpighian tubule function, revealing conserved tumor–renal crosstalk and providing a system to dissect mechanisms of organ dysfunction [187]. Such models are also employed for compound screening, including natural plant extracts, to identify interventions that inhibit stone formation or improve tubular function [188].

*Drosophila* nephrocytes, due to their ease of genetic manipulation and functional analysis, serve as an efficient platform for validating candidate genes associated with human nephropathies. Introduction of human mutant genes into *Drosophila* allows assessment of their pathological impact: rescue of nephrocyte function indicates non-pathogenicity, whereas exacerbation of defects confirms pathogenic potential [18]. This approach provides a rapid and reliable in vivo system to study gene function, disease mechanisms, and potential therapeutic interventions for renal disorders.

## 9. Genetic Conservation and Functional Genomics: Interpreting Rare Disease Variants

The widespread adoption of high-throughput sequencing has revealed a vast number of variants of uncertain significance (VUS) in clinical genetic diagnostics, creating a major bottleneck for precision medicine. *Drosophila* models, owing to their rapid generation time, low cost, and suitability for high-throughput analysis, have emerged as powerful tools to address this challenge [189]. The core approach relies on “functional rescue” or “functional mimicry” experiments. For a candidate disease-associated gene, researchers generate mutants of its *Drosophila* homolog. If the mutant exhibits phenotypes resembling the human disorder—such as neurodevelopmental defects, motor impairment, or metabolic abnormalities—introducing the wild-type human gene should rescue the phenotype. Conversely, introduction of a human gene harboring a VUS may fail to rescue or partially rescue the defect. These in vivo functional assays provide compelling evidence for reclassifying VUS as “likely pathogenic” or “likely benign” [190].

For example, VUS in the *CYFIP1* gene, implicated in neurodevelopmental disorders, was introduced into the *Drosophila Cyfip* locus via CRISPR-Cas9. The resulting mutant flies exhibited synaptic morphology and functional defects, directly confirming the pathogenicity of these variants [191,192]. Similarly, the p.A288V variant in the GABA transporter *GAT1* was overexpressed in *Drosophila*, impairing GABA uptake. This not only validated the variant’s pathogenicity but also enabled high-throughput screening of small-molecule compounds capable of restoring transporter function, providing actionable insights for personalized treatment [193]. These studies illustrate how *Drosophila* models convert abstract genetic variations into measurable, quantifiable phenotypes, significantly accelerating the functional interpretation of rare disease variants. By bridging mechanistic studies and clinical applications, these models provide a robust platform for precision medicine and translational research.

## 10. Emerging Technologies and Systems Biology: Advancing Drosophila Modeling for Precision Medicine

The forefront of *Drosophila* research is increasingly integrating cutting-edge technologies, enabling more systematic, precise, and predictive modeling of biological processes and human disease.

### 10.1. Genome-Scale and Systems Genetics

Genome-wide CRISPR screening platforms have been successfully established in *Drosophila* cell lines, facilitating systematic identification of genes essential for cell fitness under basal conditions or in response to specific drug treatments, as well as the discovery of synthetic lethal interactions. These approaches provide new avenues for cancer therapeutic target identification and functional genomics [23,194]. Complementarily, genome-scale metabolic network models have been developed for *Drosophila*, integrating thousands of metabolic reactions and associated genes. These models enable systematic simulation of metabolic responses to genetic or environmental perturbations and have been successfully applied to uncover differential metabolic pathways implicated in Parkinson’s disease, marking the emergence of *Drosophila* research into systems biology and computational modeling [195].

### 10.2. High-Resolution Imaging and Multi-Omics Integration

Advances in microfluidics and microfabrication have revolutionized *Drosophila* research, enabling high-throughput automated cultivation, behavioral monitoring, and pharmacological interventions in larvae and adults [196]. Complementing these in vivo platforms, Luhur et al. recently established continuous *Drosophila* intestinal cell lines derived from embryonic intestine through tissue-specific Ras(V12) expression. These lines can be expanded, cryopreserved, re-thawed, profiled by single-cell RNA sequencing, and, in the case of L15, grown as epithelial-polarized 3D spheroids, providing an accessible resource for multi-omic screening and intestinal biology studies [197]. Long-term two-photon imaging allows synchronized recording of neural activity and behavior in head-fixed flies navigating virtual environments, facilitating studies of prolonged processes such as learning, memory, and sleep [24]. Integration of single-cell sequencing and spatial transcriptomics has further enhanced resolution. Notably, Flysta3D-v2, a single-cell 3D spatiotemporal multi-omics atlas covering embryogenesis to pupation, systematically maps dynamic gene regulatory networks controlling cell fate and tissue development, providing a foundation for investigating disease-associated cell-type-specific changes at single-cell resolution [198].

### 10.3. Optogenetics and Precise Functional Manipulation

Optogenetic tools have also substantially expanded the experimental versatility of *Drosophila* models. Their use in flies predates many current gene-expression systems and was facilitated by the optical accessibility, small body size, and extensive GAL4/UAS resources of this organism [199,200]. Early applications relied mainly on microbial opsins such as channelrhodopsin-2 (ChR2) to activate genetically defined neurons, enabling light-controlled manipulation of sensory circuits and behavior [201]. Subsequent red-shifted tools, including ReaChR and CsChrimson, improved stimulation of freely moving animals and reduced visual-system interference, making optogenetics particularly powerful for dissecting neural circuits, locomotion, courtship, feeding, and sleep [202,203].

More recently, optogenetics has been extended from acute control of neuronal activity to precise spatiotemporal control of gene expression. ShineGAL4, a light-inducible GAL4/UAS system based on Magnet photoswitches, enables rapid and robust blue-light-dependent activation of UAS-driven transgenes across developmental stages and tissues, allowing gain- and loss-of-function manipulations at organismal, organ, and cellular levels [199]. Further refinement of this strategy has generated tissue- and cell-type-specific ShineGAL4 drivers using a split-GAL4 design, in which light-dependent assembly of GAL4 components enables inducible expression in defined tissues. Recent ShineGAL4 driver collections include lines targeting several organ systems discussed in this review, such as the fat body, gut enterocytes, oenocytes, Malpighian tubules, muscles, neurons, neuroblast lineages, and glial subtypes [204]. These developments provide a flexible framework for temporally controlled functional studies, disease modeling, and tissue-specific interrogation of pathogenic pathways in vivo.

## 11. Conclusions

In summary, *Drosophila melanogaster* has evolved far beyond a traditional genetic model. Several recent reviews have summarized the broad versatility of *Drosophila melanogaster* as a biomedical model, highlighting its genetic conservation, experimental tractability, disease-modeling capacity, and applications in drug discovery and toxicology [205]. In contrast to these general overviews, the present review adopts a tissue- and organ-centered perspective. We emphasize how conserved physiological functions and inter-organ communication in *Drosophila* provide mechanistic insight into human diseases. Furthermore, we focus on recent advances linking *Drosophila* models to gene validation, disease-associated variant interpretation, personalized therapeutic screening, and emerging technologies such as high-throughput genetic screening, single-cell/spatial multi-omics, and optogenetic control systems. Thus, this review is intended to complement existing literature by providing an organ-level and translational framework for understanding how *Drosophila* research can inform biomedical discovery and therapeutic development. Anchored in its remarkable evolutionary conservation and exceptional experimental tractability, it functions both as a “scalpel” and a “magnifying glass” for dissecting conserved mechanisms underlying complex human diseases. From proteotoxicity in neurodegenerative disorders to inter-organ communication networks in metabolic diseases, from systemic wasting in cancer cachexia to functional characterization of variants in rare genetic disorders, insights derived from this insect model have provided unique and profound perspectives across multiple biomedical fields. These foundational studies not only accelerate the discovery of conserved biological principles but also directly empower translational research by enabling construction of disease models, high-throughput genetic and pharmacological screens, and in vivo validation of clinically relevant variants. The ongoing integration of CRISPR-based gene editing, single-cell multi-omics, high-resolution live imaging, and micro-engineering technologies further enhances the precision and scope of *Drosophila* research. As our understanding of insect physiology and genomics continues to deepen, *Drosophila* will maintain an irreplaceable role in systems biology, personalized medicine, and precision disease modeling, persistently contributing its unique and powerful capabilities to the advancement of human health.

## Data Availability

No new data were created in this study.
